# Profiling SARS-CoV-2 mutation fingerprints that range from the viral pangenome to individual infection quasispecies

**DOI:** 10.1186/s13073-021-00882-2

**Published:** 2021-04-19

**Authors:** Billy T. Lau, Dmitri Pavlichin, Anna C. Hooker, Alison Almeda, Giwon Shin, Jiamin Chen, Malaya K. Sahoo, Chun Hong Huang, Benjamin A. Pinsky, Ho Joon Lee, Hanlee P. Ji

**Affiliations:** 1 Division of Oncology, Department of Medicine, Stanford University School of Medicine, 269 Campus Drive, CCSR 1120, Stanford, CA 94305-5151 USA; 2 Stanford Genome Technology Center West, Stanford University, Palo Alto, CA 94304 USA; 3 Department of Pathology, Stanford University School of Medicine, Stanford, CA 94305 USA; 4 Department of Medicine, Division of Infectious Diseases and Geographic Medicine, Stanford University School of Medicine, Stanford, CA 94305 USA

**Keywords:** COVID-19, SARS-CoV-2, Pandemic, Viral mutations, Quasispecies, Genetic fingerprints, Sequencing

## Abstract

**Background:**

The genome of SARS-CoV-2 is susceptible to mutations during viral replication due to the errors generated by RNA-dependent RNA polymerases. These mutations enable the SARS-CoV-2 to evolve into new strains. Viral quasispecies emerge from de novo mutations that occur in individual patients. In combination, these sets of viral mutations provide distinct genetic fingerprints that reveal the patterns of transmission and have utility in contact tracing.

**Methods:**

Leveraging thousands of sequenced SARS-CoV-2 genomes, we performed a viral pangenome analysis to identify conserved genomic sequences. We used a rapid and highly efficient computational approach that relies on k-mers, short tracts of sequence, instead of conventional sequence alignment. Using this method, we annotated viral mutation signatures that were associated with specific strains. Based on these highly conserved viral sequences, we developed a rapid and highly scalable targeted sequencing assay to identify mutations, detect quasispecies variants, and identify mutation signatures from patients. These results were compared to the pangenome genetic fingerprints.

**Results:**

We built a k-mer index for thousands of SARS-CoV-2 genomes and identified conserved genomics regions and landscape of mutations across thousands of virus genomes. We delineated mutation profiles spanning common genetic fingerprints (the combination of mutations in a viral assembly) and a combination of mutations that appear in only a small number of patients. We developed a targeted sequencing assay by selecting primers from the conserved viral genome regions to flank frequent mutations. Using a cohort of 100 SARS-CoV-2 clinical samples, we identified genetic fingerprints consisting of strain-specific mutations seen across populations and de novo quasispecies mutations localized to individual infections. We compared the mutation profiles of viral samples undergoing analysis with the features of the pangenome.

**Conclusions:**

We conducted an analysis for viral mutation profiles that provide the basis of genetic fingerprints. Our study linked pangenome analysis with targeted deep sequenced SARS-CoV-2 clinical samples. We identified quasispecies mutations occurring within individual patients and determined their general prevalence when compared to over 70,000 other strains. Analysis of these genetic fingerprints may provide a way of conducting molecular contact tracing.

## Background

**Fig. 1:**
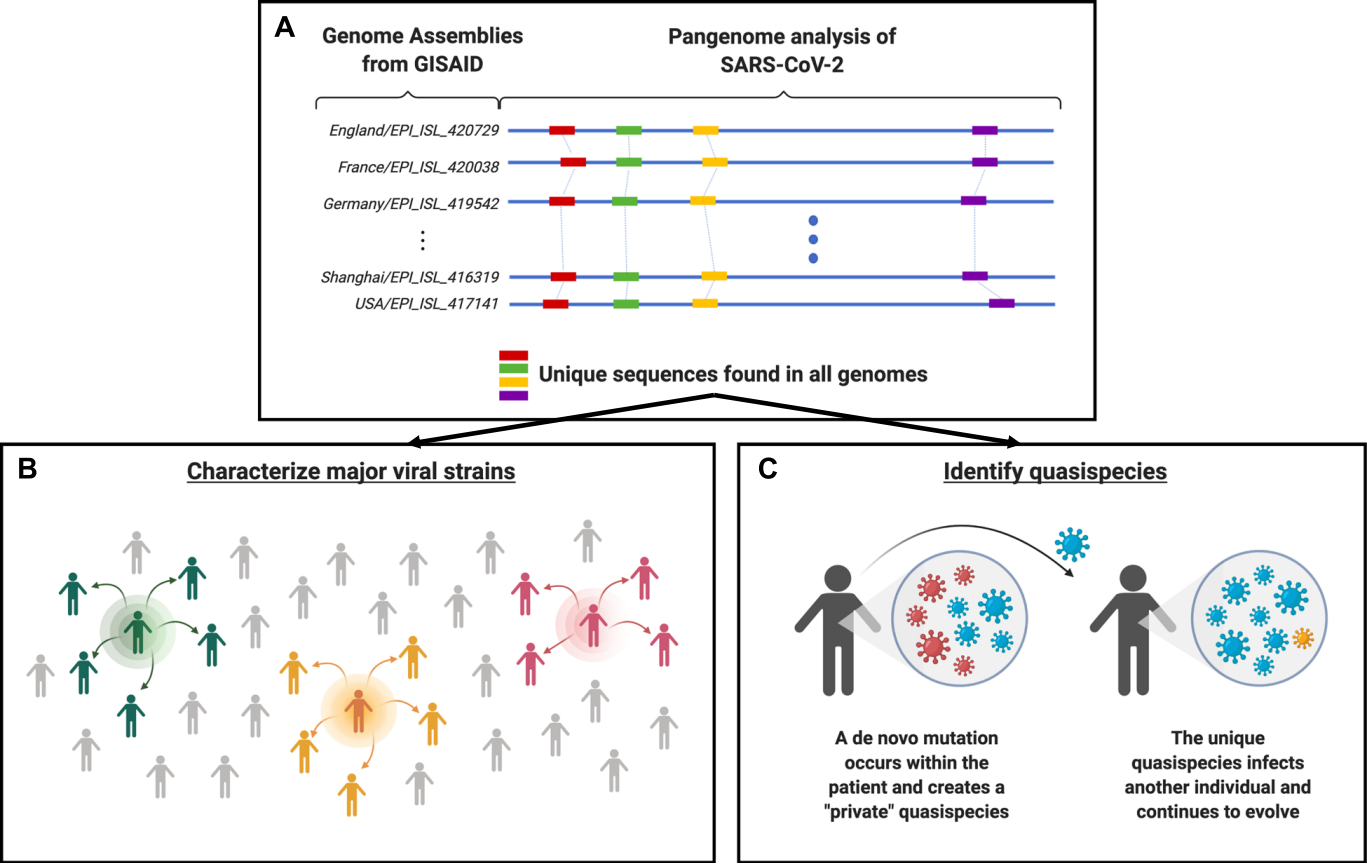
Framework for identifying population-level SARS-CoV-2 strains and lower frequency quasispecies through pangenome analysis. **a** GISAID currently has thousands of SARS-CoV-2 genomic sequences banked. By analyzing large numbers of viral genomes together (blue lines), one can pinpoint sequences that are present only once in a given genome but also occur consistently across all genomes (red, green, yellow, and purple bars). These unique and conserved sequences can be used for a number of research and clinical sequencing applications. **b** This knowledge can fuel epidemiological studies and allow scientists to characterize major SARS-CoV-2 strains. The green, orange, and pink figures represent contagious individuals who contribute to the transmission of a virus within a population. **c** The targeted sequencing enables the detection of low-frequency quasispecies that are created through de novo mutations within an individual. The mutation profile from pangenome analysis allow us to examine whether these mutations create a unique genetic profile that can be traced to and across individuals

The etiological agent of the COVID-19 pandemic is the SARS-CoV-2 coronavirus, encoded by a single-stranded RNA molecule [1]. Given its airborne transmission and adaptation for highly efficient human-to-human transmission, this virus has rapidly spread across disparate geographic regions and infected diverse populations [2]. An important genetic feature of nucleic acid replication is the accumulation of mutations. There are two general categories of mutations based on their frequency among infected individuals (Fig. 1). Strain-level mutations are found in a relatively high frequency among affected populations and provide viral genetic fingerprints from which one can trace the routes of transmission across broad geographic regions. This genetic information is useful in contact tracing for super-spreader events, where a given viral strain is introduced among a group of exposed individuals [3]. On a more granular scale, individual patients with active infections also show evidence of de novo mutations that occur as the virus replicates within the individual [4, 5]. These novel mutations define subclonal quasispecies that are private to an individual [6]. Occurring at a significantly lower population frequency, quasispecies-level mutations are frequently present in only a fraction of the total viral load of an infected individual and may be specific to a given patient. Any of these viral subpopulations can infect another individual. If a quasispecies unique to an individual is transmitted to another, this information could be used in characterizing transmission patterns of infection.

RNA-dependent RNA polymerases (RdRp) are the major source of mutations in coronaviruses. Although RNA polymerases are essential for viral replication, this class of polymerases has high error rates, leading to the accumulation of mutations in the viral genome over time [7]. For coronaviruses, estimates of RNA polymerase mutation rates range from 10^− 4^ to 10^− 6^ mutations per a given nucleotide [8]. Moreover, coronavirus genomes undergo both homologous and nonhomologous recombination which provides additional genetic variation for fueling viral evolution [9]. Although SARS-CoV-2 mutation rates are lower than viruses such as human immunodeficiency virus or influenza [10, 11], the frequency of replication-based mutations is high enough for genetic fingerprinting analysis and its related applications.

Currently, the most common diagnostic methods detect viral RNA, viral antigens, or antibodies produced by the host during an immune response [12]. These molecular detection assays do not provide information about specific viral mutations. Thus, next-generation sequencing (NGS) is required to identify viral mutations. NGS provides comparable detection sensitivity to the gold standard PCR assays while simultaneously providing viral genome sequence. In addition, sequencing studies of SARS-CoV-2 have generated complete viral genome assemblies, where the entire end-to-end sequence of a viral isolate is reconstructed [13].

With the growing number of sequenced SARS-CoV-2 genomes, researchers have identified numerous mutations indicative of different phylogenetic lineages, otherwise known as clades, and related to specific strains transmitted in the population [14]. Genetic information from these public datasets can be leveraged to trace the spread of infection across different populations. Specifically, one can compare genetic fingerprints using mutation profiles from different viral data sets to map the spread of infections [3, 15], identify virulence factors, and study features of viral evolution in populations. In these studies, genome assemblies and phylogenetic analyses from a densely sampled cohort were used to determine how SARS-CoV-2 was introduced into a new geographic region. Prospectively, the study of these mutations also provides a foundation for designing future vaccines and developing drugs for antiviral targets [16].

To date, there are tens of thousands of samples and matched genome assemblies for SARS-CoV-2 [17]. With this data, it becomes feasible to conduct “pangenome” studies that simultaneously analyze all of the available viral genome assemblies. The analysis of the SARS-CoV-2 pangenome is useful for determining the common sequence features of all viruses as well as the high frequency mutations that distinguish the viral species most prevalent in an infected population [18]. However, analyzing thousands of viral genome assemblies is time consuming. Among the challenges, there is a lack of common sequence criteria for defining conserved versus hypervariable genomic sequences prone to mutation. Distinguishing between these features is important for understanding the viral evolution, spread, and designing future molecular assays that are specific to SARS-CoV-2.

This study had two objectives relevant for viral genetic fingerprinting (Fig. 1). The first goal was developing a rapid, efficient method for identifying conserved sequences and mutation profiles across thousands of different viral SARS-CoV-2 genomes in parallel. The second goal was focused on leveraging our results from pangenome analysis to develop a targeted deep sequencing assay. We used it to identify genetic fingerprints characteristic of viral quasispecies from clinical samples and compared these results to the pangenome.

Most SARS-CoV-2 genomic studies have relied on conventional sequence alignment, which is slow and inefficient when applied on a scale of thousands of genome sequences [19–21]. To overcome this issue and facilitate viral pangenome studies, we developed a rapid computational approach to identify conserved sequences across any number of SARS-CoV-2 genome sequences. This method does not use sequence alignment. Rather, we employ k-mers, short sequences of tens of bases, for annotating and querying the viral genome sequences and genetic variation. This feature has computational advantages for streamlining the analysis of large number of genome sequences and comparing the features of assemblies [20]. We applied our k-mer approach to find the highly conserved sequences across thousands of SARS-CoV-2 genome assemblies and other viral genome sequence data sets. This process provided the rapid identification of conserved regions of the genome and enabled us to index mutations across thousands of viral genomes.

Based on our identification of conserved regions of the SARS-CoV-2 pangenome, we developed a sequencing assay to identify novel mutations. Rather than covering the entire viral genome, we used highly conserved sequences as primer sites that flank highly variable genome regions. The assay provides very deep sequencing coverage on the average of thousands of reads per a given base. We analyzed a series of contrived samples, artificial viral admixtures, and clinical samples. These results from our pilot study showed that one can achieve both high sensitivity and specificity for detecting SARS-CoV-2 with deep targeted sequencing. We detected mutations in the SARS-CoV-2 genome at varying allelic fractions representative of emerging quasispecies. Importantly, when comparing our results with viral mutations from public data sets of viral genomes, our analysis revealed private mutations representing quasispecies unique to a single infected individual. Our targeted assay, utilizing only six amplicons to target 40% of the viral genome, is flexible towards any region of interest. This multiplexed targeted next-generation sequencing assay demonstrated potential for massive scalability required for population studies.

## Methods

### Viral samples

This study was conducted in compliance with the Helsinki Declaration. The Institutional Review Board at Stanford University School of Medicine approved the study protocol. A total of 100 extracted RNA specimens from clinical nasopharyngeal swabs were obtained from the Stanford University Clinical Virology Lab (Stanford University, Stanford, CA). All samples were anonymous and had no identifiers. The specimens consisted of 30 positives and 70 negatives that had been clinically tested at the Stanford Clinical Virology Laboratory with the SARS-CoV-2 detection RT-qPCR assay (FDA EUA200036).

### Saliva sample collection and processing

Saliva samples were obtained from two (*n* = 2) healthy donors with no symptoms of respiratory infection (presumed SARS-CoV-2 negative) at Stanford University School of Medicine after obtaining their written informed consent. Saliva samples were collected using the OMNIgene ORAL OM-505 kit (DNA Genotek, Canada) according to the manufacturer’s instructions. Cells were lysed using the buffer provided in the caps of the sample collection tubes. Lysed saliva samples were inactivated at 65 °C for 15 min prior to RNA extraction. We extracted RNA using the Maxwell 16 Viral Total Nucleic Acid Purification Kit (Promega, Madison, WI) according to the manufacturer instructions. RNA samples were eluted with 40 μL of RNAse-free water. Human RNA extracted from saliva samples of SARS-CoV-2-negative donors was quantified using the Qubit RNA HS Assay Kit (Thermo Fisher Scientific, Waltham, MA) and stored at − 80 °C.

### Control samples

As an analytical positive control, we spiked genomic RNA from SARS-Related Coronavirus 2, Isolate USA-WA1/2020 (ATCC, Manassas, VA), into two saliva samples from healthy individuals at Stanford University. To assess the limit of detection, we generated a series of 12 contrived samples at total inputs ranging from 1 × 10^3^ to 0 genome copies. We generated an additional set of 3 SARS-CoV-2 dilutions at the lowest inputs (e.g., 2 copies, 1 copy, and 0 copies) for downstream cDNA synthesis to confirm the limit of detection for assay.

### Admixture analysis for detection of sub-clonal mutation allelic fraction

We created an additional set of 12 contrived samples using genomic RNA from two previously characterized SARS-Related Coronavirus 2 strains: Isolate USA-WA1/2020 and Isolate Hong Kong/VM20001061/2020 (ATCC, Manassas, VA). These two strains were spiked into the same PCR-confirmed SARS-CoV-2-negative human RNA samples at different relative fractions. All samples had the same total concentration of viral RNA (1000 copies).

### Genome sequences of SARS-CoV-2, other human viruses and bacteria

We accessed the data available from three different sources: SARS-CoV-2 genomes from Global Initiative on Sharing All Influenza Data (GISAID) [18]; other human coronaviruses from Virus Pathogen Resource (ViPR) [22]; and lastly, other human viruses and bacteria sequences from the National Center for Biotechnology Information (NCBI) (Additional file 1; Table S1) [23]. According to GISAID, the definition of “complete” means the genome is greater than 29 kb and “high-coverage” means only select entries with 1% N’s and < 0.05% unique amino acid mutations (not seen in other sequences in database) and no insertion/deletion unless verified by the submitter. We also identified 447 human coronavirus genomes by selecting “host: human,” “complete genome,” and “date: up to 2019 Oct” from ViPR. Furthermore, we obtained 804 virus genomes with human host from NCBI viral genomes.

We identified and removed coronaviruses from the compiled 804 virus genomes. As a result, we had our own list of the virus genomes consisting of 42 influenza viruses, and 320 human viruses. “Respiratory syncytial virus” was added to our human virus list in acknowledgement of the existence of this virus in the FDA’s cross-reactivity list. With this, all viruses on the FDA’s cross-reactivity list were included in our list. Additionally, we retrieved and added the reference genomes of 27 bacteria in the FDA’s cross-reactivity list. We used *Rothia mucilaginosa* DY-18 searched by “*Stomatococcus mucilaginosus*,” which is the new name of *Staphylococcus salivarius*. NCBI selected *Pneumocystis jirovecii* RU7 (assembly Pneu_jiro_RU7_V2) as reference genomes for *Pneumocystis jirovecii*. The accession numbers of all SARS-CoV-2 genomes from GISAID and all other sequences used in this study can be downloaded from our GitHub repository: https://github.com/compbio/sars-cov-2-mutation-fingerprints [24].

### Metadata of 25-mers present in SARS-CoV-2 genomes and other genomes of interest

We generated a searchable index of location metadata for all 25-mers found in 3968 SARS-CoV-2 genomes. Our scripts and indices are also available at our GitHub repository https://github.com/compbio/sars-cov-2-mutation-fingerprints [24]. We included all of the 25-mers from 837 human viruses, bacteria, and the human genome reference GRCh38 (Additional file 1; Table S1). The metadata provides the locations of all distinct canonical k-mers in all sequences of interest, such as the 3968 SARS-CoV-2 genomes included in this analysis. A canonical k-mer is the lexicographically smaller sequence of a k-mer and its reverse complement. For instance, the metadata for the 25-mer “AGGGACTATTCCCACCCAAGAATAG” in the SARS-CoV-2 genomes contains the following information: sequence ID, position, and strand, appearing in three sequences (distinct entries separated by semicolons):

*USA/CruiseA14/EPI_ISL_413619/2020-02-25, 11742-11766,+; Shangrao/JX1974/EPI_ISL_421258/2020-02-08,11718-11742, +; USA/TX_2020/EPI_ISL_419561/2020-02-29,11742-11766,+;*

We generated a table of k-mer counts within all SARS-CoV-2 genomes in the 4K dataset, which can be viewed as a matrix with one row for each genome, one column for each distinct k-mer, and with values indicating the count of a k-mer in a genome. We utilized this matrix to conduct downstream analyses such as primer selection and computation of variant frequencies. We provide this index and a command-line interface that makes it easy to query this index (e.g., for listing all candidate primer pairs targeting a particular genomic region) at https://github.com/compbio/sars-cov-2-mutation-fingerprints [24]. The command-line interface is written in the Julia programming language [25, 26], but does not require the user to program in Julia to be used, for easier interoperation with other software. Our analysis was run on an AMD EPYC 7501 32-Core Processor, 503 GiB, Linux version 4.4.0-184-generic #214-Ubuntu SMP (Advanced Micro Devices, Inc., Santa Clara, CA).

Intuitively, a k-mer is “specific” to a “target” set of genomes with respect to another, “off-target” set of genomes if the k-mer occurs in many of the target genomes and does not occur in any of the off-target genomes, even allowing for a few mismatching base pairs in the off-target set. Moreover, a k-mer is “unique” within a genome if it occurs exactly once within that genome. A k-mer is “X% conserved” within a set of genomes if it occurs in at least X% of those genomes. “Anchor” k-mers are both 100% conserved and unique within each genome. A k-mer is “X% specific with up to M mismatches” to a target set of genomes G with respect to off-target set of genomes G’ if it is X% conserved in G and does not occur in any genome in G’ even if we allow up to M mismatching base pairs.

In the example above, a 25-mer appears in three SARS-CoV-2 genomes at the coordinates printed. These coordinates are relative to the respective sample genomes. Crucially, our method supports fast, approximate search, necessary to satisfy constraints of maximum 80% similarity to other human viruses/bacteria and 90% similarity to GRCh38 as outlined by the FDA’s SARS-CoV-2 detection assay EUA criteria. Our 25-mer index stores location metadata for all 25-mers in a list of genomes and also allows approximate lookup by allowing up to M mismatching base pairs when querying the set of locations at which a 25-mer appears. For example, the 25-mer AATTGTACTGTTTTTAACAAAGCTT is conserved and unique among the 3968 SARS-CoV-2 sequences but is not specific to SARS-CoV-2 because it occurs within 2 mismatches at 2 locations in GRCh38 (see below; dots indicate base pairs matching GRCh38, and capital letters denote mismatches).

*‘seq’: ‘.........T.......A...….’,‘chr1’, ‘pos’:‘183137629-183137653’, ‘strand’:‘-’,*

*‘seq’: ‘....A.........C..........’, ‘chr16’, ‘pos’:‘457072-457096’, ‘strand’:‘-’*

This enables us to identify 25-mers that have no approximate matches (1) up to 4 mismatched base pairs with the genomes of all human coronaviruses, other human viruses, influenza, and the 27 bacteria included in the FDA’s SARS-CoV-2 detection assay cross-reactivity list, and (2) up to 2 mismatched base pairs with the human genome (GRCh38).

We also constructed a k-mer index containing all k-mers induced by all possible single-substitution mutations, two previously reported deletions, and multi-nucleotides substitutions in the N gene. To make this index, for each variation we took the set of k-mers from the SARS-CoV-2 reference genome that overlaps the reference allele position—the “reference set,” modified these k-mers to instead contain the alternate allele—the “alternate set,” and then associated each of these modified k-mers with metadata describing the variation. For example, suppose that (1) *k* = 3, (2) our variant is the substitution from A to C at position 1000, and (3) the reference sequence from position 998 to 1002 is CGATT. Then the reference set of 3-mers is (CG**A**, G**A**T, **A**TT) (reference allele in bold), the alternate set of 3-mers is (CG**C**, G**C**T, **C**TT) (alternate allele in bold), and our k-mer index would map each of the alternate 3-mers to a description of this variant. An “alternate” k-mer can be associated with multiple distinct mutations, but for *k* = 25 we found that most of the alternate 25-mers were associated uniquely with exactly one mutation. Any 25-mers that were not associated with exactly one mutation were removed from the index. This 25-mer index allows us to survey efficiently mutations in viral assemblies from GISAID without using a sequence alignment. The k-mer index file is available for download at our GitHub repository https://github.com/compbio/sars-cov-2-mutation-fingerprints [24]. In other words, we obtain the frequency of a particular mutation simply by counting the number of genomes that have the mutation-specific 25-mers. In general, k-mers belonging to all classes of mutations can be detected, but our annotation of complex variants such as indels and multi-nucleotide substitutions is limited to reported sets because the enumeration of all possible indels and multi-nucleotide substitutions can be arbitrarily large.

### Quantitative PCR

We used the TaqPath™ 1-Step RT-qPCR assay (Thermo Fisher Scientific, Waltham, MA) on the StepOnePlus Real Time Quantitative PCR instrument (Applied Biosystems, Foster City, CA) as an additional validation test on the contrived samples. Briefly, RNA samples were reverse transcribed to cDNA and then subjected to 45 cycles of quantitative PCR according to the following recommended conditions: UNG incubation at 25 °C for 2 min, reverse transcription incubation at 50 °C for 15 min, enzyme activation at 95 °C for 2 min, 45 cycles of amplification consisting of denaturation at 95 °C for 3 s followed by annealing/extension at 60 °C for 30 s, and a final infinite holding step at 4 °C. We used previously described primers (CDC 2019-nCoV Real-Time RT-PCR Diagnostic Panel) and 6-carboxyfluorescein (FAM)-labeled hydrolysis probes targeting three regions of the SARS-CoV-2 nucleocapsid protein (N) gene [27]. We also used additional primer probe set targeted a human housekeeping (*RPP30*) gene as an internal/extraction control. The primers and probes used for both the SARS-CoV-2 targets and the human *RPP30* targets were identical to those described in the CDC nCoV-19 assay [27].

### Digital PCR

We measured the concentrations of both human (*RPP30*) cDNA and SARS-CoV-2 cDNA in the contrived samples by performing multiplexed droplet digital PCR (ddPCR) using ddPCR Supermix for Probes (no dUTP) (Bio-Rad, Hercules, CA), the CDC nCoV-19 N1 assay (IDT, Newark, NJ), and a commercially available human *RPP30* genomic DNA assay (Bio-Rad, Hercules, CA) on the QX200 Droplet Digital PCR System (Bio-Rad, Hercules, CA). The N1 assay included a FAM-labeled hydrolysis probe and the *RPP30* assay included a HEX-labeled hydrolysis probe. Viral and human genomic targets were amplified following droplet generation according to the following recommended cycling conditions: incubation at 25 °C for 3 min, enzyme activation at 95 °C for 10 min, 40 cycles of denaturation at 94 °C for 30 s followed by annealing/extension at 60 °C for 1 min, enzyme deactivation at 98 °C for 10 min, and a final infinite holding step at 4 °C. Following amplification, droplets were analyzed using the QX200 Droplet Reader and QuantaSoft Software (Bio-Rad, Hercules, CA).

### Identifying sequencing primers

We generated a list of candidate primer pairs for targeted sequencing. Let G_cov2 denote the set of 3968 SARS-CoV-2 GISAID genomes, let G_other denote the set of viral and bacterial genomes, and let G_human be the human reference genome GRCh38. A candidate primer pair consists of two 25-mers denoted x (forward primer) and y (reverse primer) that satisfy four properties: conservation and uniqueness, specificity, positional constraints, and compositional constraints. To be conserved and unique, both x and y must appear in all 3968 SARS-CoV-2 sequences exactly once. In order to be specific, at least x or y in a pair must not occur in G_other, even allowing for up to 4 out of 25 mismatched base pairs (i.e., maximum 80% similarity to any 25-mer in G_cov2). Additionally, x or y must not occur in G_human even allowing for up to 2 out of 25 mismatched base pairs. If the primer pair meets the requirements for positional constraints, the maximum distance between x and y in any sequence in G_cov2 must be within a heuristic limit of 2500 base pairs, not including the combined 50 base pairs of x and y. We chose a size of 2.5 kb in order to reduce the need for extended long-range PCR optimization. Having long amplicons covering significant portions of SARS-CoV-2 also decreases the complexity of multiplex PCR amplification of many short amplicons. Finally, the compositional constraints mean that the GC content of x and y may differ by 2 out of 25 base pairs at a maximum.

We identified 67,478 candidate primer pairs that met the above criteria. The entire list of candidate primer pairs can be found at our GitHub repository https://github.com/compbio/sars-cov-2-mutation-fingerprints [24]. Primer pairs were selected from the list of candidates, and each amplicon was validated individually using simplex PCR (as in “Targeted sequencing of SARS-CoV-2,” below). Individual primers from neighboring candidates were also manually evaluated, which increased the maximum amplicon size to 2.67 kb.

We selected six primer pairs to target the SARS-CoV-2 genome for our PCR workflow (Additional file 1; Table S4). In addition, we selected *RPP30* as a human transcript control, which was used in the Centers for Disease Control and Prevention’s (CDC) RT-PCR diagnostic testing. To ensure all copies of *RPP30* were amplified, we designed primers for both human cDNA and genomic DNA. We selected highly conserved sequences such that genetic variation among individuals would not affect the ability of the primers to anneal to their complement. Specifically, we selected conserved regions that flank regions of high variability, according to frequencies in the Genome Aggregation Database (gnomAD) [28]. For human DNA, we chose two forward and reverse primer pairs. The first primer pair sequences *RPP30* across exon 1 to produce an amplicon of 0.476 kb. The second primer pair sequences a region between exons 6 and 7 to produce an amplicon of 1.277 kb. Within the amplicon of the second primer pair, there are three regions with high variability. If the variability of each region is 50% such that one out of two individuals is likely to have a single nucleotide polymorphism, then there are 8 unique amplicons that can arise from that primer pair. Each individual therefore has a slightly distinct amplicon that can be used to differentiate patient samples within a pool.

### Targeted sequencing of SARS-CoV-2

Based on the final set of primer pairs (Additional file 1; Table S4), we developed a two-step multiplex PCR protocol for the reverse transcription and PCR amplification of SARS-CoV-2 targets from both the clinical and contrived samples. Briefly, cDNA was synthesized from RNA samples using random hexamer priming and ProtoScript II First Strand cDNA Synthesis Kit (New England Biolabs, Ispwich, MA) according to the manufacturer’s instructions. We generated amplicons from cDNA samples using the Titanium Taq PCR Kit (TaKaRa Bio, Japan) according to the following recommended cycling conditions: enzyme activation at 95 °C for 1 min, 40 cycles of denaturation at 95 °C for 30 s followed by annealing/extension at 68 °C for 2.5 min, final extension at 68 °C for 3 min, and a final infinite holding step at 4 °C. One microliter of cDNA from contrived samples was used for the PCR reaction, and 5 μl was used for the patient samples to maximize the amount of genomic material to be amplified. We used a panel of six SARS-CoV-2-specific primers sets targeting non-overlapping regions ranging from 1 to 2.67 kb in length across the viral genome, and a primer set targeted the human *RPP30* gene as an extraction control (Additional file 1; Table S4). PCR was performed on a Veriti Thermal Cycler (Applied Biosystems, Foster City, CA).

We performed massively parallel library preparation using a pooled tagmentation approach with the plexWell™ 384 kit (seqWell, Beverly, MA). Briefly, this library preparation workflow consists of a two-step transposase-based process referred to as tagmentation, where sequencing adapters are randomly inserted into the PCR amplicons DNA by transposition. The first tagmentation step incorporates well-specific Illumina i7-Read 2 barcodes. After pooling, a second tagmentation step incorporating a plate-specific Illumina i5-Read 1 barcode finishes the library. After PCR amplification, we performed a 1:10 dilution of the PCR plate for use with the plexWell protocol per manufacturer’s instructions, with the only adjustment being a final magnetic bead cleanup using a ratio of 0.9X beads rather than 0.75X to enrich for shorter fragments. After library preparation, the sample was quantified on a Qubit 4 Fluorometer (Thermo Fisher Scientific, Waltham, MA), visualized on a 2% E-Gel EX cartridge (Thermo Fisher Scientific, Waltham, MA), and loaded on an iSeq 100 reagent cartridge (Illumina, San Diego, CA) with 5% PhiX library control v3 (Illumina, San Diego, CA) spike-in with 151 bp paired-end reads and 8 bp dual indexing.

### Bioinformatic sequencing analysis

Raw sequence data underwent base calling and demultiplexing using bcl2fastq v2.20 (Illumina, San Diego, CA). Reads were aligned using bwa (mem algorithm; v0.7.17) and processed into bam files using samtools (v1.10) [29]. As a reference genome, we generated a merged reference by concatenating GRCh38 and the ancestral SARS-CoV-2 reference sequence (NC_045512.2) into a single FASTA file and creating a new bwa reference. Reads aligning to either the human or viral genome were counted by using the command “samtools idxstats,” and per-base coverage metrics were analyzed using bedtools (v2.29) using the “bedtools coverage -d” command, selecting only for the SARS-CoV-2 genome.

For identification of variants in clinical samples, we performed variant calling using Sentieon (v201808.08) with the reference genome of SARS-CoV-2 (NC_045512.2) and the reference human genome (GRCh38). First, we preprocessed FASTQ files containing raw sequence data with quality control using the default setting of fastp. Second, the paired-end alignments from the filtered reads against were implemented by the Sentieon bwa binary. Next, deduplication and realignment around indels were performed on each sample: “sentieon driver --algo Dedup and --algo Realigner”. Fourth, we used the haplotype caller setting to conduct variant calling with base quality recalibration, which was derived from the recalibrated bam files: “sentieon driver --algo Haplotyper –annotation QD,MQ, MQRankSum,ReadPosRankSum,FS, SOR,DP”. Finally, variants consistent between two or more replicates from each respective patient sample were considered real mutations for each sample and were included in our downstream mutation profile analyses. Downstream analysis used R for generating plots and statistical calculations.

## Results

### k-mer analytics across a SARS-CoV-2 pangenome

To identify conserved regions across thousands of SARS-CoV-2 genome assemblies, we developed a computational workflow that analyzes k-mer sequences (Fig. 1a). We developed our approach using 3968 SARS-CoV-2 genome assemblies, each representing a different viral sample (Additional file 1; Table S1, Additional file 2; Table S10). The sequence assemblies were obtained from GISAID. We built a series of k-mer indices across the entire viral data set. For any given genome, an individual k-mer is derived from single-base increments along the length of the viral genome (“Methods”). Thus, in a genome with a length of 30 kb, the approximate size of the SARS-CoV-2 genome, there are ~ 30,000 k-mers. If all viral genomes had the same sequence, this number would not change.

The length of the k-mer sequences was another important variable that we considered. We evaluated k-mers ranging from 21 to 29 bases, the typical length of primers for molecular assays. We chose odd lengths to prevent issues associated with searching for reverse complement sequences. Many of viral k-mer sequences could be identified in the human genome within relatively short edit distances when the length was set at 21 or 23 bases (Additional file 1; Table S2). Increasing k-mer length is a trade-off between increasing specificity for differentiating SARS-CoV-2 sequences from other viral, bacterial, and human genome sequences, and reducing the total number of possible conserved and unique k-mers that can be represented in the SARS-CoV-2 genome. Balancing these factors, we chose a k-mer length of 25 bases (“25-mer”).

We generated an index of 25-mers from all viral assemblies by associating each 25-mer with the following metadata: (1) IDs of all viral assemblies containing the 25-mer, (2) start position coordinates within each viral assembly containing the 25-mer, and (3) the frequency of each 25-mer within each viral assembly. We identified a total of 94,402 different k-mers from our viral assembly data set. This high number of different k-mers directly reflects viral genome assemblies with mutations. Namely, a variation in sequence such as a mutation generates novel k-mers, thus leading to an increase in the total k-mer number. Without any mutations, we would expect the counts of k-mers around 30,000, which is size of reference SARS-CoV-2 genomes. At this baseline state without additional filtering, the k-mer index corresponded to a matrix M with 94,402 columns (one for each 25-mer), 3968 rows (one for each SARS-CoV-2 genome), and with elements M [*i*,*j*] being the number of times the *j*th 25-mer appears in the *i*th genome. The k-mer index also contains the locations of each k-mer in each genome, expressed in the local coordinate system per a given genome as metadata. As we demonstrate later, this index enables to generate annotations and compare mutations among different viral genomes very efficiently.

We identified all of the 25-mers that were present only once within an individual viral assembly (“Methods”). In other words, our definition of a unique k-mer is one where the sequence is found only once in that individual SARS-CoV-2 genome (Table 1). However, the same unique k-mer can also be found in other SARS-CoV-2 assemblies so long as its uniqueness is maintained per a given viral genome (Fig. 1b). This simple definition provided us with a way of measuring conservation across different viruses and employing a conservation score. We identified 1977 k-mers which had the property of being unique among all of the k-mers for a given viral genome and where the same 25-mer sequence demonstrated this feature across all of the genomes in our data set (k-mer conservation at 100%). We referred to these conserved sequences as “anchor k-mers” given their properties of both uniqueness and conservation across the pangenome. Additional details about the derivation and properties of the anchor k-mers are described in the “Methods”.

**Table 1:** Identification of unique k-mers from viral assemblies

Unique 25-mers found among the SARS-CoV-23,968 genomes: 0 present, 1 = absent	AATGTATAGGGTCAGCACAAAAAAT	AGATAATAGATGGTAGAATGTAAAA	AGGGACTATTCCCACCCAAGAATAG	TAAGACTGTATGCGGTGTGTACATA	TCGTAACAATTAGTATGCCAGCAAA	TGCACCAAGTGACATAGTGTAGGCA	TTGGGTTCCACGTGCTAGCGCTAAC	TTTCACTGCACTTTGGAAAGTAACA
England/EPI_ISL_420729	0	1	0	1	1	1	0	1
France/EPI_ISL_420038	1	1	0	1	1	1	1	1
Germany/EPI_ISL_419542	0	1	0	1	1	1	1	0
Netherlands/EPI_ISL_415482	0	1	0	1	0	1	1	1
Shanghai/EPI_ISL_416319	0	1	0	0	1	1	1	1
USA/EPI_ISL_413619	0	1	1	1	1	1	1	1
USA/EPI_ISL_420793	0	1	0	1	1	1	1	1
USA/EPI_ISL_417141	0	1	0	1	1	1	1	1
•	•	•	•	•	•	•	•	•
3968 SARS-CoV-2 samples	2	3968	3	3966	3958	3968	3960	3965
SARS-CoV-2100% conserved	No	Yes	No	No	No	Yes	No	No
Human genome (GRCh38)	0	0	0	0	0	0	0	1
42 influenza sequences	0	0	1	0	0	0	0	0
89 bacteria sequences	0	0	0	0	0	0	0	1
447 other human-host coronaviruses	0	0	0	10	0	0	0	0
321 other human-host viruses	0	0	1	0	0	0	0	0
SARS-CoV-2 specific	Yes	Yes	No	No	Yes	Yes	Yes	No

Our approach was conducted independent of a coordinate system for any given genome and therefore was independent of the choice of a specific SARS-CoV-2 reference. If we conducted this study with an alignment method, comparisons of viral genome assemblies would have scaled exponentially as the number of samples inevitably increased. On the other hand, our method of characterizing the viral pangenome scales linearly and therefore is computationally efficient. Our analysis took a total of 5 s using 32 cores on a server to index approximately 4000 viral genome assemblies using the k-mer approach. In comparison, using multiple sequence alignment with Clustal Omega [30] took 712 s for only 10 genomes; with Kalign [31], we measured 423 s for 40 genomes. Extrapolating these metrics means multiple sequence alignment would require a minimum of 12 h for 4000 viral genomes when neglecting exponential scaling.

### Conservation and mutation landscapes of SARS-CoV-2 resolved with k-mer indexing

**Fig. 2:**
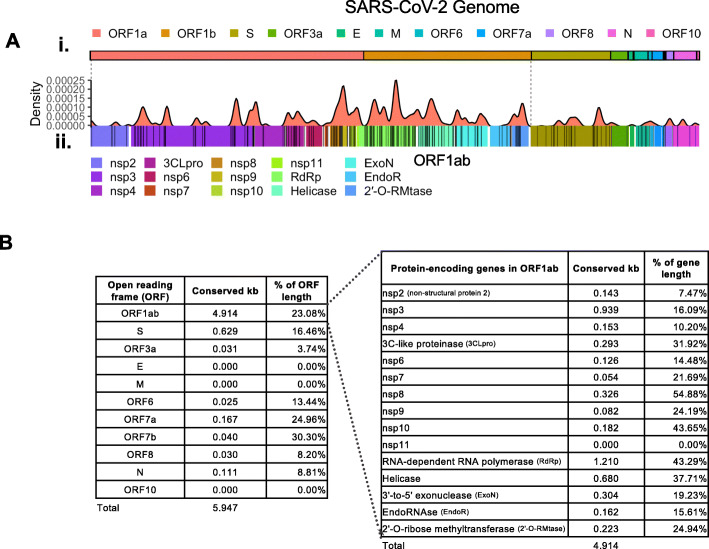
SARS-CoV-2 landscape of conserved regional sequences. A total of 1977 conserved 25-mers (“anchor 25-mers”) were identified from the 3968 SARS-CoV-2 genome sequences included in the k-mer analysis. **a** Distribution of anchor 25-mers across the (i) SARS-CoV-2 genome. Individual anchor 25-mers are shown as (ii) black lines with their overall density. Conserved regions consist of either discrete or overlapping anchor 25-mers and vary in total length (black rug plot). The density of conservation refers to the total number of base pairs comprising anchor 25-mers within each consecutive 100-bp window across the genome (red kernel density plot). **b** The total number of conserved base pairs within each given region of the genome varies across ORFs and genes

We grouped the 1977 anchor 25-mers based on overlapping sequence. As result, we identified 166 highly conserved regions across the 3968 viral assemblies (Fig. 2). The number of overlapping anchor 25-mers in these conserved regions ranges from 1 to 98 with median of 8. The total size of conserved sequences was 5.947 kb, which is 19.8% of the 29.9 kb reference genome. To display the extent of conserved sequences, we plotted the position of these sequences across the length of a reference viral genome NC_045512.2 for gene and protein annotations (Fig. 2a). The gene with the highest number of conserved 25-mers was *orf1ab* (Fig. 2b), which is also the largest viral gene. We considered the gene length in the context of the entire genome and normalized the representation as a fraction of the total sequence. By correcting for sequence length, we found that *orf7a* and *orf7b* were the most highly conserved at 24.96% and 30.3%, respectively.

The *orf1ab* sequence encodes multiple proteins (Fig. 2b). Interestingly, three protein-coding regions within *orf1ab* had high percentage of conserved sequences: 54.88% for nsp8; 43.65% for nsp10; 43.29% for RdRp. In contrast, the *E* gene did not overlap with any conserved regions. This result suggests that the *E* gene is more likely to subject to significant mutations during the course of viral evolution.

From our initial pangenome dataset of 3968 SARS-CoV-2 genomes, we identified 2346 mutations occurring in at least one SARS-CoV-2 genome assembly (Additional file 3; Table S12). Focusing on point mutations, 1005 (42.8%) were non-synonymous mutations, and 1577 mutations (63.9%) were unique to a single isolate. Thirty-eight mutations were found in more than 1% of genomes, while 15 mutations were found in more than 5% of genomes. The most common mutations found in more than 40% of genomes were as follows: (1) 14408 C>T; RdRp P323L (45.7%), 2) 3037 C>T; nsp3 F106 (45.3%), 3) 23403 A>G; S D614G, and 4) 241 C>T; 5′ UTR (45.2%).

**Fig. 3:**
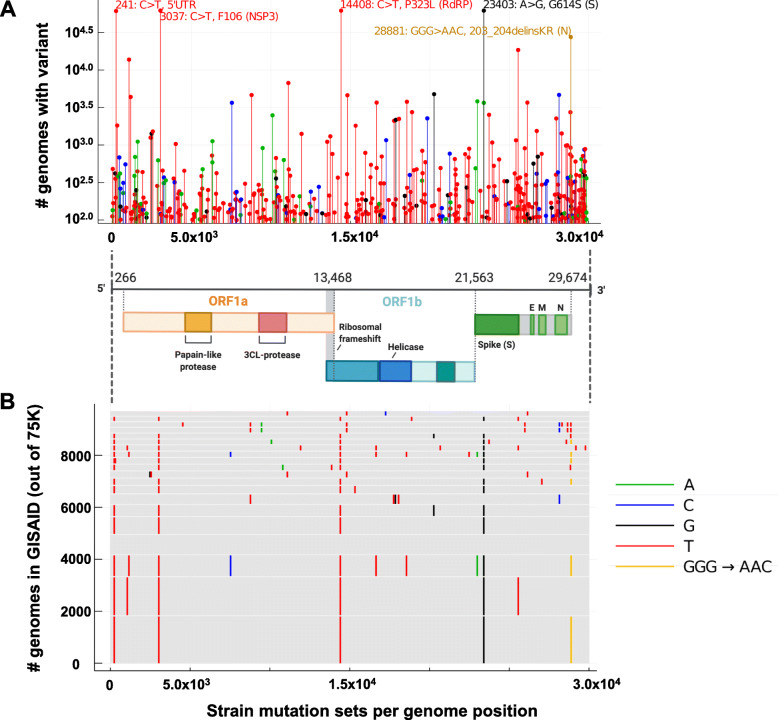
Individual and joint frequencies of mutations in 75,681 SARS-CoV-2 genomes. **a** Mutations occurring in at least 100 of the 75,681 SARS-CoV-2 genomes, with top 5 most frequent mutations annotated. Colors indicate the variant allele (see legend). **b** Top 20 most frequent co-occurring sets of mutations (“fingerprints”) among 75,681 GISAID genomes. Each gray rectangle corresponds to one fingerprint, with vertical line segments located at the positions of the variants in that fingerprint, and with line segment colors indicating the variant alleles (see legend). The height of each rectangle is equal to the number of GISAID genomes with the associated fingerprint, so the single most frequent fingerprint is the bottommost rectangle. The 4th most frequent fingerprint (4th rectangle from the bottom) has no variants relative to the SARS-CoV-2 reference genome

We further conducted an expanded pangenome analysis by examining an additional set of 75,681 complete and high-coverage viral genomes from GISAID (downloaded on 09/23/2020). These mutations were distinguished by patterns of unique 25-mer sequences (Figure S1) and were incorporated into our k-mer mutation index. We identified 20,671 mutations from at least one viral genome assembly; (1) 15,914 of these mutations (21%) were found in less than 0.001% of genomes and (2) 8071 (10.6%) were unique to a single sample (Fig. 3a, Additional file 3; Table S13). On average, each viral genome contains 8.61 mutations with a standard deviation of 3.45.

From this expanded pangenome analysis, we identified mutation profiles that were highly specific viral identifiers, or genetic “fingerprints,” based on their relatively low frequency among these samples. Among the notable characteristics, 12,552 (60.7%) mutations were nonsynonymous, 7234 (35%) were synonymous, and 885 (4.3%) mutations occurred within non-coding regions, such as the 5′ UTR and the 3′ UTR. Fifty-seven mutations were found in greater than 1% of assemblies while 14 variants were found in greater than 5% of assemblies. We found that *RdRp* had the largest number of mutations that occurred in more than 1% of assemblies (7 out of 57 mutations). The most common mutations we observed were as follows: 62,018 (81.95%) for 3037C>T (F106) in the nsp3 protein; 61,993 (81.91%) for the 23404A>G (D614G) in the *S* gene; 61,920 (81.82%) for 14408C>T (P323L) in the RdRp protein (Fig. 3a).

Using our k-mer mutation index, we identified the twenty most common combination profiles of mutations, or “fingerprints,” among the 75,681 genome assemblies that we examined (Fig. 3b). As shown in Fig. 3b, the top 20 SARS-CoV-2 genetic fingerprints represented the mutation profile of approximately 11.6% of viral assemblies. The remaining 88.4% had more discrete sets of mutations, suggesting that these signatures occurred less frequently across the viral genomes included in our analysis. Notably, the fourth most frequent fingerprint (occurring in approximately 1% of assemblies included in our analysis) had no mutations relative to the SARS-CoV-2 reference genome (Fig. 3b). The overall majority of viruses included in our analyses had at least one viral genetic signature with the vast majority occurring at low frequencies.

### Quantitative measurement of SARS-CoV-2 with deep targeted sequencing

Based on our pangenome results, we designed a targeted sequencing assay using the highly conserved sequences from anchor 25-mers. Our goal was to develop a robust amplification assay covering a wide portion of the viral genome but not necessarily the entire viral genome. This latter point was important, seeing that we intended to generate sequencing coverage in the thousands at a reasonable sequencing cost and with a minimal number of amplicons.

As part of the amplicon design, we considered the following parameters for primer sequences: (1) specific only to SARS-CoV-2; (2) present in highly conserved regions per our pangenome analysis; (3) flanking non-conserved regions; (4) having DNA properties such as GC content that facilitated multiplex PCR; (5) minimizing the number of amplicons to reduce multiplexing artifacts. Based on these combined parameters, we anticipated that our primer sets had a very high probability of amplifying any SARS-CoV-2 genomes regardless of the location of any mutations. As illustrated in Fig. 2, we found a total of 1977 anchor 25-mers, all being highly conserved across nearly 4000 viral genomes. Interestingly, when we examined the ARTIC primers which are commonly used for viral genome sequencing [32], only 57% of the primers appeared in at least 99% of the genomes in the 75,681 k GISAID dataset.

We followed the Food and Drug Administration’s (FDA) Emergency Use Authorization guidelines for designing SARS-CoV-2 detection assays [33]. The FDA’s criteria include testing for cross-reactivity against other viral, bacterial, and human genomes (Additional file 1; Table S3). To reduce the chance of off-target amplification, we eliminated any candidate primer sequences up to an edit distance of four present in other human coronavirus, human viruses, and bacteria. Likewise, we used the same criteria for sequences that were found in the human genome and excluded any candidate primer sequences within an edit distance of two (“Methods”).

**Fig. 4:**
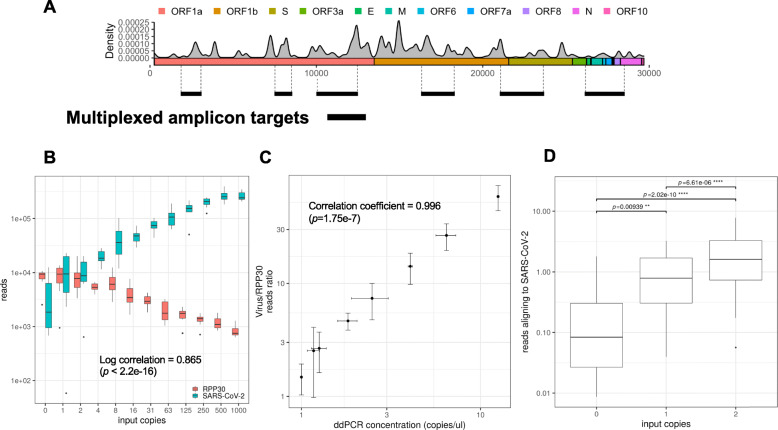
Deep targeted sequencing of SARS-CoV-2 by multiplex amplicon generation. **a** Amplicon targets. Six amplicons (dark line) ranging from 1 to 2.6 kb were used to target approximately 40% of the SARS-CoV-2 genome. The density of anchor 25-mers indicating high-sequence similarity across GISAID isolates is also shown. **b** Dynamic range of SARS-CoV-2 sequencing. Next-generation sequencing libraries were created from the SARS-CoV-2 amplicons as well as targeting the *RPP30* control gene. Libraries consisted of serial dilutions (*N* = 8 replicates) of purified SARS-CoV-2 genomic RNA into total nucleic acid derived from a COVID-negative saliva sample. Reads aligning to either *RPP30* or SARS-CoV-2 are shown, alongside with the log-log correlation to the number of input copies of virus in each sample. **c** Correlation with digital PCR. At each dilution used for sequencing, a portion of cDNA was used for digital PCR (*N* = 3 replicates), targeting the N1 gene. The corresponding concentration derived from digital PCR is shown alongside reads aligning to virus versus the *RPP30* control (correlation coefficient = 0.996, *p* < 1.75e-7). **d** Limit of detection testing. A larger number of replicates (*N* = 32) was performed at 0, 1, and 2 input viral copies to assess the limit of detection of the assay. Shown are the reads aligning to both SARS-CoV-2 and the *RPP30* control gene. Significance values (FDR-corrected) which test against the negative control (0 copies) are shown

This multiplexed assay had six amplicons targeting the more variable regions of the SARS-CoV-2 genome, for a total coverage of approximately 39.9% of the viral genome (Fig. 4a; Additional file 1; Table S4). Amplicon sizes ranged from 1 up to 2.67 kb. In addition, we amplified a region in human gene *RPP30* as a positive control against technical problems that may occur during RNA extraction. Polymorphisms detected in this gene also serve as a control against potential sample swaps or contamination.

First, we used randomly primed reverse transcription of viral and human RNA. This step was followed by a multiplexed PCR amplification. Afterwards, to generate Illumina-compatible sequencing libraries we used a two-step transposase approach where the first step incorporates a unique DNA barcode among 96 wells and the second pooled reaction adds a plate-specific barcode (“Methods”). For the first step, the amount of tagmentation conducted by transposase is limiting for any given reaction volume. Thus, sequencing libraries are automatically normalized prior to loading onto a sequencing instrument, reducing the total hands-on operational time. With these new features, our experimental approach was highly scalable, enabling the generation of hundreds of normalized sequencing libraries in a single day without the need for robotic automation.

We tested this sequencing assay on cDNA generated from a serial dilution of twelve different concentrations of commercially available SARS-CoV-2 genomic RNA (Fig. 4). The SARS-CoV-2 RNA serial dilution was spiked into total nucleic acid extracted from human saliva. The human RNA sample was SARS-CoV-2 negative as per qPCR. The range of SARS-CoV-2 concentrations spanned three orders of magnitude from 1000 total copies to 1 total copy per a given sample (Additional file 1; Table S5). We generated and sequenced eight technical replicates per concentration from the cDNA samples. Following Illumina sequencing, the data was processed and aligned to both the human genome (GRCh38) and the SARS-CoV-2 reference NC_045512.2. Overall, the coverage in viral regions was over 1000X with high viral inputs with over 95% of target regions sequenced, but declined as viral inputs decreased towards single viral copies (Additional file 4; Table S14). There was a high correlation between sequence reads aligning to the SARS-CoV-2 genome compared to the amount of viral RNA (Fig. 4b) with a log-log correlation coefficient of 0.865 (*p* < 2.2e−16)*.* In parallel, as the viral input decreased, the proportional number of *RRP30* reads increased. Here, we found that deep targeted sequencing enabled sensitive detection of SARS-CoV-2.

To validate the quantitative performance of the sequencing assay, we performed digital PCR (dPCR) on the same batch of cDNAs used in the SARS-CoV-2 dilution series (Fig. 4c). Digital PCR provides absolute quantitative measurement of RNA templates at near single-molecule resolution. We used a set of PCR primers for the N1 region and a TaqMan-based oligonucleotide probe to detect the presence of template in each droplet (“Methods”). We observed a high concordance between digital PCR and sequencing (Pearson correlation coefficient = 0.996; *p* = 1.75e−7; log-transformed values), supporting equivalent sensitivity between the two methods (Fig. 4d). Overall, we demonstrated sequencing-based viral detection at a resolution as low as a single copy per reaction.

We further validated the lower detection threshold of sensitivity of our sequencing assay. For this experiment, we used the cDNA samples with absolute quantitation from dPCR. We generated 32 amplicon replicates from cDNA samples containing two, one, and zero SARS-CoV-2 genomic copies (Fig. 4d). We observed statistically significant differences between one and zero input copies (*p* = 0.0094; one-sided FDR-corrected *t*-test), suggesting that this sequencing assay may have more sensitive detection compared to other molecular diagnostic assays for SARS-CoV-2 detection [34].

### Detection of viral mutation allelic fractions

**Fig. 5:**
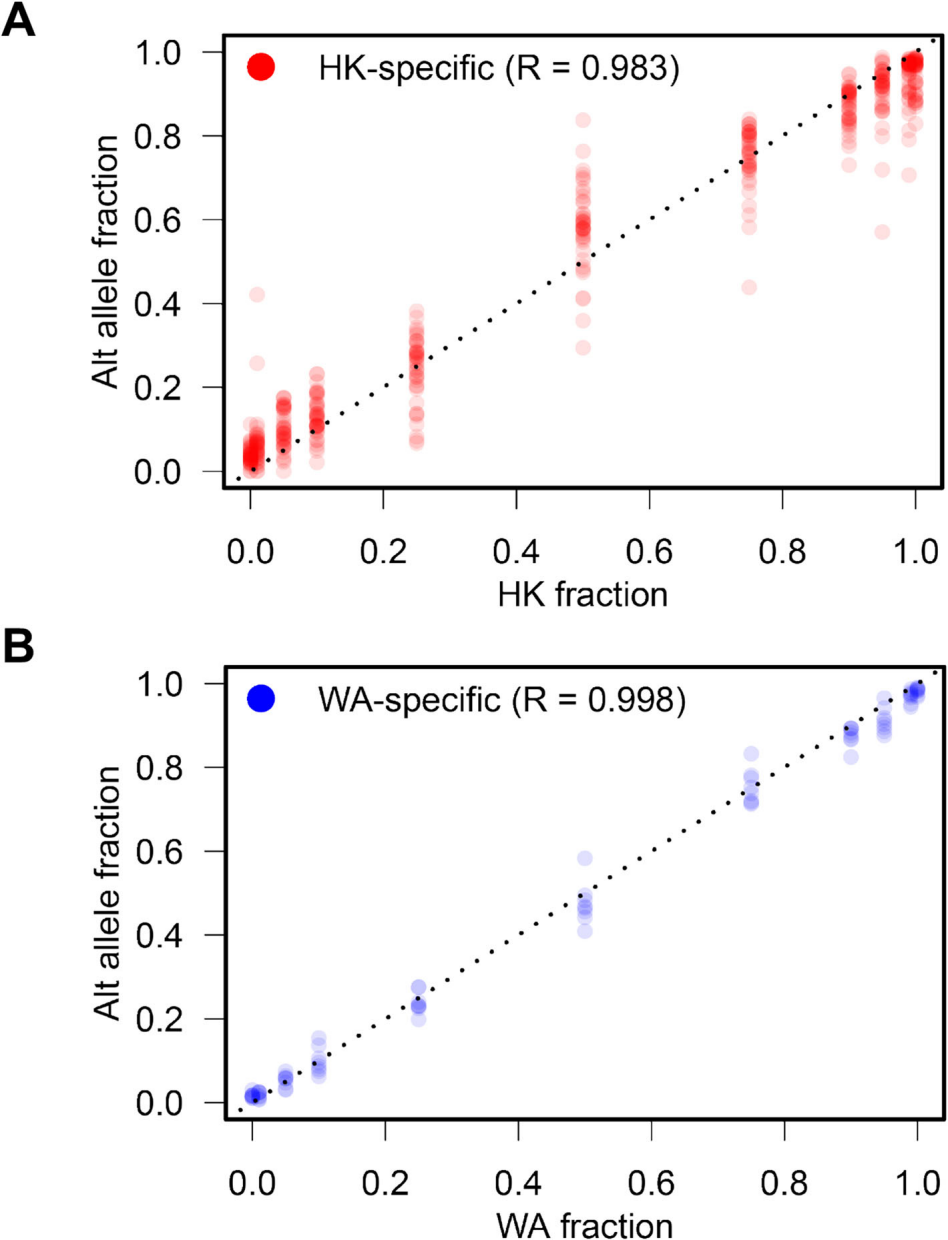
Fraction of alternative alleles specific to viral strains in admixtures. The allelic fraction of exclusive mutations, measured from all the eight replications, was compared against the ratio of viral strains, separately for the Hong Kong (**a**) and the Washington (**b**) strains. The admixtures contain 0%, 1%, 5%, 10%, 25%, 50%, 75%, 90%, 95%, 99%, or 100% of either strain (the *x*-axes). The allelic fraction of exclusive mutations was plotted on the *y*-axes. Areas of the plot with many overlapping points appear as dark clusters, and the dotted diagonal line indicates 1:1 concordance. For both the strains, the Pearson’s correlations (R) are indicated

We validated our sequencing assay for detecting mutations at low read frequency and resolving genetic fingerprints. We generated admixtures of USA-WA1/2020 and Hong Kong/VM20001061/2020 purified SARS-CoV-2 RNA (ATCC, Manassas, VA) to test the capability of the k-mer-based mutation index to distinguish mutations among different viral strains. Within the targeted regions that we sequenced, the Hong Kong strain had a total of six exclusive mutations and the Washington state strain had one exclusive mutation (Additional file 1; Table S6). We prepared viral RNA admixtures with different relative fractions of each strain for a total input of 1000 copies, which were spiked into human nucleic acid. The admixture ranged from a high of 99% to a low value of 1% for a given viral strain component. Eight technical replicates of each admixture were prepared and sequenced in parallel. The average sequencing coverage for all the replicates was in the thousands (Additional file 4; Table S14). Using the published assembly sequences of each strain and their associated mutations, we determined the read coverage of each mutation base (Additional file 1; Table S7). We plotted with the empirical value versus the expected allelic fraction (Fig. 5). In the case of the mutations with the Hong Kong strain, the correlation coefficient was 0.983. For the Washington strain, the correlation coefficient was 0.998. Our results showed that the measured mutation allelic fraction per our sequencing data highly correlated with the theoretical admixture fraction. Moreover, mutations expected to be present at a 1% allelic fraction were detected across all replicates. This result indicates that sensitive detection of low allelic fraction mutations was feasible. At the time of the study, the Hong Kong and Washington viral samples were the only commercially available sources of purified RNA that was suitable for genomic mixture analysis. We analyzed genomic assemblies from two additional commercially available strains, hCoV-19/Germany/BavPat1/2020 and 2019-nCoV/Italy-INMI1 (ATCC). Our additional mutation analysis revealed at least one distinguishing mutation per strain that could be used to detect and characterize the given strain. Therefore, our admixture analysis can broadly be applied to other SARS-CoV-2 strain standards provided the defining mutations are covered by our designed amplicons.

### Viral quasispecies analysis of a SARS-CoV-2 patient cohort

We examined a set of SARS-CoV-2 clinical samples that included both positive and negative samples previously evaluated with a RT-qPCR assay (FDA EUA200036) approved by the US Food and Drug Administration (FDA). We sequenced a total of 100 extracted RNA clinical specimens from nasopharyngeal swabs obtained from patients tested at the Stanford Clinical Virology lab. The specimens consisted of 30 positive samples and 70 negative samples (Additional file 1; Table S8, Additional file 4; Table S14). A total of three cDNA technical replicates were made from each of the 30 positive RNA samples for immediate downstream use in PCR amplicon generation and sequencing library preparation. Data is available through the National Institutes of Health’s Short Read Archive [35].

Overall, each sample yielded high quality reads, of which Q30 scores were over 90%. In addition, 99% of the reads aligned to either the human or SARS-CoV-2 genome (Additional file 4; Table S14). We observed that some samples had substantially lower sequencing yield. Upon further investigation, these samples had low numbers of reads in both viral and human targets across replicates, indicating low viral RNA yield from nasopharyngeal swabbing or sample extraction. Viral sequencing coverage varied from less than 100X to greater than 10,000X, indicating large-scale variation in viral load across patients.

**Fig. 6:**
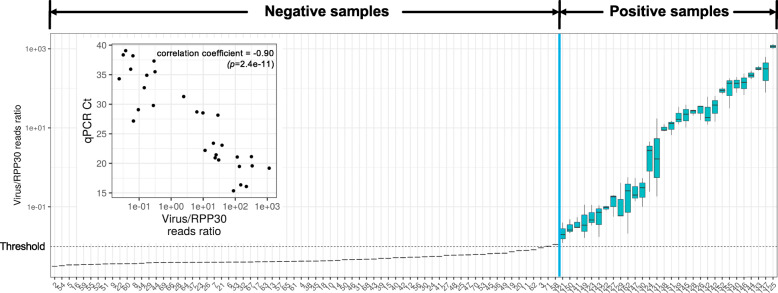
Targeted sequencing of SARS-CoV-2 of clinical samples. Targeted sequencing was performed on a total of 100 clinical samples (30 positive and 70 negative) derived from nasopharyngeal swabs. The ratio of reads aligning to viral and *RPP30* sequences is displayed, with samples shown in increasing order of the ratio of reads aligning to SARS-CoV-2 to *RPP30*. The positive samples (blue; *N* = 3) were found to be higher than the reads ratio of negative samples by setting a threshold of greater than three times greater than the standard deviation away from the mean of the negative samples (dashed line). Inset: Correlation of viral reads ratio to qPCR *C*_T_ values as derived from clinical tests (correlation coefficient = − 0.90, *p* = 2.4e−11)

While developing the assay, we observed that the proportional number of reads aligning to either SARS-CoV-2 or *RPP30* was dependent on the number of viral copies loaded. Therefore, we utilized the virus-to-*RPP30* read ratio as a normalization metric to assess performance and detection (Fig. 6). We found that this metric had a high correlation to the *C*_T_ values from qPCR diagnostic test (Pearson correlation coefficient of − 0.90, *p* = 2.4e−11) (Fig. 6). Previously found to be negative for SARS-CoV-2 by qPCR, these 70 clinical samples provided us with a calibration threshold for determining a positive case. We determined the average read ratio and standard deviation to be 0.00494 and 0.00163, respectively. We set our threshold at three standard deviations (0.00488) above the average to detect the presence of SARS-CoV-2 reads. Based on this definition, the sequencing results were positive for 30 out of 30 samples previously diagnosed by qPCR. All negative samples had a read ratio of less than 2%, well below the read ratio of the positive samples. To assess our criteria for separating positive and negative samples, we used the same threshold calculations with a random subset of negative samples (50%, *N* = 35) resulted in a false positive rate of 0.03 and a false negative rate of zero (*N* = 100 trials). A small proportion of viral reads were noted in the negative controls and samples. These viral reads were an artifact in which sample indexes are swapped among multiplexed libraries when using the Illumina platform [36]. To quantify this effect, we leveraged the admixture control experiments; in sequence data of only the Hong Kong strain, we measured the amount of reference nucleotides where we expected to observe only the variant allele. Using a cutoff depth of 500 at variant positions, we measured the index hopping rate to be 2.4%.

**Fig. 7:**
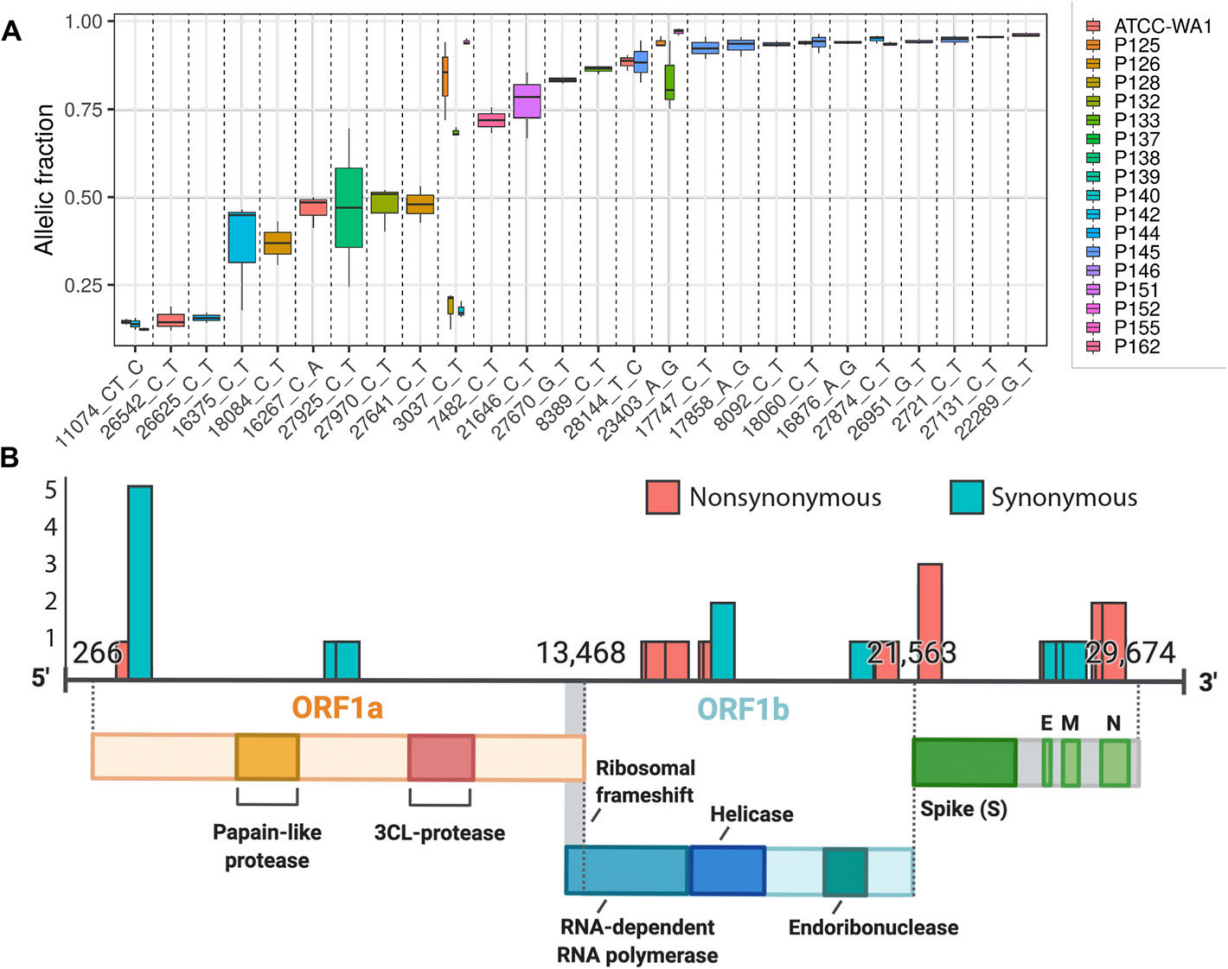
Quantifying viral quasispecies signatures in clinical samples. **a** Allelic fraction across mutations. Each boxplot represents the distribution of allele fractions for each viral sample at a particular viral genome position. Some mutations were found in multiple patients and have multiple boxplots. **b** Distribution of SNVs across the viral genome. The number of mutations found at each position, as well as the type of variant (synonymous vs. nonsynonymous) is shown

After sequence data processing, we identified mutations from the 30 COVID-19 positive clinical samples and a control SARS-CoV-2 isolate (“Methods”). We identified a total of 37 mutations across 26 positions from 16 out of the 30 clinical samples (Table 2). The remaining 14 samples showed no variant differences from the reference assembly NC_045512.2. We observed a broad range of allelic fractions from mutations in our clinical sample set, ranging from less than 10%, indicative of subclonal mutations, to strain-specific mutations with allelic fractions greater than 90% for a given viral sample (Fig. 7a). We observed 25 positions with substitutions (16 missense and 9 silent) (Fig. 7b). One deletion was observed at a single site for one sample. Mutations leading to substitutions were noted at 20 of the 26 total positions. These mutations were unique to an individual sample. The remaining six substitutions were found in more than one sample (Table 2). Among our samples, six mutations were also found in with high frequency (greater than 1000 samples) in different data sets such as the WHO SARS-CoV-2 collection (*N* = 10,022) and the GISAID (*N* = 75,681) collection. Four mutations were observed in the SARS-CoV-2 genomic RNA isolate from Washington which we used as a positive control. Interestingly, two of these four mutations were shared with one patient sample (P145) and two were private to the positive control.

**Table 2:** Observed SARS-CoV-2 mutations in clinical samples

Mutation	Gene / open reading frame	Translational change	Sample frequency ***(n = 30)***	Samples	GISAID frequency ***n = 75,681)***
2721 C>T	Nsp3	A1V	1	P145	7
3037 C>T	Nsp3	Synonymous	5	P133, P152, P128, P140, P125	62,018
7482 C>T	Nsp3	S1588L	1	P162	10
8092 C>T	Nsp3	Synonymous	1	P145	42
8389 C>T	Nsp3	Synonymous	1	P133	154
11074 CT>C	NA	Microsatellite	3	P155, P128, P142	NA
16267 C>A	Helicase	Q11K	1	ATCC_WA-01	NA
16375 C>T	Helicase	P47S	1	P142	16
16876 A>G	Helicase	T214A	1	P132	0
17747 C>T	Helicase	P504L	1	P145	2104
17858 A>G	Helicase	Y541C	1	P145	2167
18060 C>T	3′ to 5′ exonuclease	Synonymous	2	ATCC_WA-01, P145	2233
18084 C>T	3′ to 5′ exonuclease	Synonymous	1	P126	4
21646 C>T	S	Synonymous	1	P151	257
22289 G>T	S	A243S	1	P152	15
23403 A>G	S	D614G	3	P152, P125, P133	61,993
26542 C>T	M	T7I	1	ATCC_WA-01	13
26625 C>T	M	Synonymous	1	P144	86
26951 G>T	M	Synonymous	1	P146	44
27131 C>T	M	Synonymous	1	P137	16
27641 C>T	ORF7a	S83L	1	P126	31
27670 G>T	ORF7a	V93F	1	P139	35
27874 C>T	ORF7b	T40I	2	P144, P146	40
27925 C>T	ORF8	T11I	1	P138	22
27970 C>T	ORF8	T26I	1	P132	66
28144 T>C	ORF8	L84S	2	P145, ATCC_WA-01	4655

We observed five mutations, each private to a single sample, in the helicase gene from three clinical samples (Table 2). One sample (P145) had two mutations in the helicase gene and the other two (P142 and P132) had only one mutation. The two helicase mutations (17747 C>T and 17858 A>G) observed in sample P145 were reported in 2104 (2.78%) and 2167 (2.86%), respectively, out of the 75,681 GISAID genomes included in our analysis. The other mutations observed in the helicase gene were present in less than 0.03% of the 75,000 genomes annotated in GISAID genomes.

We identified three nonsynonymous mutations in *orf7* and three nonsynonymous mutations in *orf8* from the clinical specimens. All but one (27874 C>T in *orf7b*) were private to their respective samples (Table 2). Two of the mutations in *orf7* were in *orf7a* (P126 and P139) and one was in *orf7b* (P144 and 146). All three *orf7* mutations were present in less than 0.1% of the SARS-CoV-2 GISAID genomes included in our pangenome analysis. In *orf8*, one mutation (28144 T>C) was identified in a clinical sample (P145), present in the positive control strain (ATCC_WA-01) and observed in 4655 genomes, or 6.15% per GISAID. The other mutations in *orf8* were seen in frequencies less than 0.1% in the GISAID pangenome analysis (Table 2, Additional file 1; Table S9). Interestingly, all of the mutations observed in both *orf7* and *orf8* among our clinical sample set are expected to result in significant changes to the amino acid side chain polarity (Table 2, Additional file 1; Table S9). For example, three of the six mutations observed between *orf7* and *orf8* (27874 C>T in samples P144 and P146, 27925 C>T in sample P138, 27970 C>T in P132) lead to an amino acid change from threonine to isoleucine, which constitutes a major change in sidechain polarity.

**Fig. 8:**
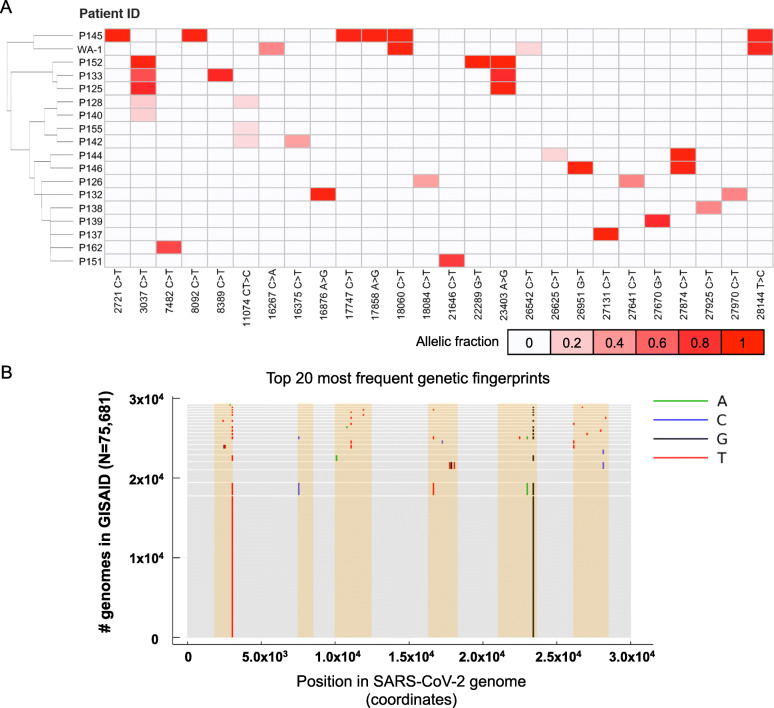
Heat map of allelic fractions in positive clinical samples. **a** Rows and columns indicate samples and the mutations, respectively. Result from a hierarchical clustering is shown as dendrogram. To best represent evolutionary relationship among samples, the clustering was based on the genotype itself (i.e., mutation or no mutation) instead of the allelic fraction values. The mutations are sorted by the genomic position. **b** Top 20 most frequent co-occurring sets of mutations (“fingerprints”) among 75,681 GISAID genomes, with variants restricted to lie inside the regions of the SARS-CoV-2 genome that we sequenced. Each gray rectangle corresponds to one fingerprint, with vertical line segments located at the positions of the mutation in that fingerprint, and with line segment colors indicating the mutation alleles. The height of each rectangle is equal to the number of GISAID genomes with the associated fingerprint, so the single most frequent fingerprint is the bottommost rectangle. The 3rd most frequent fingerprint (3rd rectangle from the bottom) has no variants relative to the SARS-CoV-2 reference genome within the sequenced regions

Several studies have illustrated that the previously observed D614G variant in the spike protein potentially increases the infectivity of SARS-CoV-2 as indicated by both in vitro viability experiments in cell culture and lower *C*_T_ values in clinical settings [37, 38]. Our results are concordant with this observation, as samples containing the D614G variant had higher proportions of sequences aligning to SARS-CoV-2 (Fig. 6 and Fig. 8). This observation was also confirmed from diagnostic PCR, where patients with D614G mutations had mean *C*_T_ values of 18.56 ± 2.94 (*N* = 3) versus mean *C*_T_ values of 27.9 ± 7.24 (*N* = 27) (Additional file 1; Table S8).

### Comparisons of SARS-CoV-2 genetic fingerprints

We compared our mutation profiles among the 17 patient samples with strain-specific or quasispecies-level mutations representative of subclonal viral populations. Hierarchical clustering of mutations points out some potential group structure (Fig. 8). While some individual mutations were shared between two or more samples, no two samples had the same mutation profile. In other words, each patient had their own unique genetic viral fingerprint with no direct match among our set of clinical samples. Notably, we observed private mutations for each sample. Three clinical viral samples had mutations which occurred in less than 0.01% of 75k GISAID viral samples.

As we described previously, we constructed a k-mer mutation index database in which individual mutations were represented for 75,681 viral samples from GISAID. These mutations had passed quality control criteria (“Methods”). The k-mer index facilitated rapid pairwise similarity calculations among our samples versus those from the GISAID (Table 3). When making comparisons from our clinical cohort with this expanded set of samples, we used two different criteria between our viral sample set that underwent deep sequencing versus the mutation signatures from GISAID (Table 3). First, we applied strict matching criteria such that a mutation set per a given Stanford clinical sample exactly matched the set of another sample annotated in GISAID. When using the strict matching criteria, there were seven samples that were unique and not found among the 75,681 GISAID samples. In addition, there were four patients (P137, P139, P146, P152) with viral samples which did match those in GISAID but were present at a frequency of less than 0.0001%.

**Table 3:** Viral mutation fingerprint comparisons

Patient	Private mutation^**1**^	Reported mutations^**2**^	Strict matching # genomes^**3**^	Relaxed matching # genomes^**4**^	Ratio relaxed criteria
P125		3037C>T, **23403A>G**	17,851	43,943	5.806345E−01
P126	8084C>T	**27641C>T**	0	0	0.000000E+00
P128		**3037C>T**	66	61,952	8.185938E−01
P132	16876A>G	**27970C>T**	0	0	0.000000E+00
P133		**3037C>T**, 8389C>T, **23403A>**	37	115	1.519536E−03
P137		27131C>T	1	15	1.982003E−04
P138		**27925C>T**	0	22	2.906938E−04
P139		**27670G>T**	1	34	4.492541E−04
P140		**3037C>T**	66	61,952	8.185938E−01
P142		**16375C>T**	0	16	2.114137E−04
P144		26625C>T, **27874C>T**	0	0	0.000000E+00
P145	**2721C>T**	8092C>T, **17747C>T, 17858A>** 18060C>T, **28144T>C**	0	0	0.000000E+00
P146		26951G>T, **27874C>T**	3	0	0.000000E+00
P151		21646C>T	0	257	3.395833E−03
P152		3037C>T, 22289G>T, 23403A>	4	9	1.189202E−04
P162		**7482C>T**	0	10	1.321336E−04

^1^Private mutations refer to those with a frequency less than 0.001%

^2^Mutation in bold are non-synonymous

^3^Strictly refers to other samples with the exact same number of mutations

^4^Relaxed refers to samples that are inclusive for these mutations but may have others

Given that we only had partial genome coverage, we considered a second criterion in assessing the genetic fingerprints observed in patient samples (Table 3). We had initially required that the mutation signatures from the clinical samples have an exact mutation set match. Under our new relaxed matching criteria, we also included GISAID samples with mutations additional to those identified in our sequencing study. Given that the alignments downloaded from GISAID were obtained via whole genome sequencing, this allowed us to include samples with mutations outside of the regions we targeted.

When we considered the relaxed criteria, there were four samples which had no matches with GISAID (P126, P132, P144, P145). Nine samples (P133, P137, P138, P139, P142, P146, P151, P152) had matches to viral sequences in GISAID, but when considering them as a subtotal, these sets of mutations occurred at a frequency of less than 0.001% among the GISAID genomes included in our analysis. There were three patients (P125, P128, P140) in which, when using the relaxed matching criteria, the frequency of matching GISAID samples ranged from 5.8 to 8.2%. Overall, a significant fraction of patients (13/30) had viral mutation signatures which were relatively unique, even when using this relaxed criterion.

We evaluated the frequency of specific genetic fingerprints. The P125 viral sample matched the most common GISAID fingerprint consisting of 3037 C>T and 23403 A>G (Fig. 3b), indicative of a common viral strain. The P155 viral sample matched the 3rd most common fingerprint in which there are no variants distinct from the NC_045512.2 reference which originated from Wuhan. The P125 viral sample had a common mutation signature with 17,851 exact matches (Fig. 8b), and P155 has 1587 exact matches. Seven viral samples did not match any of the top 20 most common strain fingerprints. This included viral samples from P128, P133, P137, P139, P140, P146, and P152 which had a range of 1 to 66 matches. These samples had relatively unique fingerprints given that they were present in less than 0.001% in the larger GISAID data set. Overall, our results suggest that unique and relatively rare viral mutation fingerprints can be identified among a significant number of patients.

## Discussion

With the rapid transmission of SARS-CoV-2 around the world, different strains have emerged as defined by the propagation of viruses with new mutations. Our study leveraged a systematic k-mer-based analysis of the SARS-CoV-2 to identify critical regions of viral sequence conservation and provide mutation indexes across tens of thousands of viral sequences from GISAID. We used this sequence analysis to develop a robust targeted sequencing assay to detect viral mutations with high sensitivity by focusing specifically on those regions identified as hypervariable in our k-mer analysis. We subsequently compared mutation profiles observed in the clinical sample set to those reported in GISAID. Based on the results of our study, this approach could be leveraged to provide a highly scalable and integrated framework for identifying viral genetic fingerprints among patients that are relatively unique. Importantly, this process may facilitate molecular contact tracing where one compares viral mutation fingerprints among infected individuals, which can bolster our understanding of how transmission events occur throughout the global population. This notion is broadly supported by population-scale sequencing initiatives such as GISAID to characterize SARS-CoV-2 and enumerate lineages [39], and further builds upon it at the individual level by measuring viral genetic fingerprints at clonal and subclonal resolution. Recently, genomes representing the novel SARS-CoV-2 lineage B.1.1.7 have been observed around the world. This novel strain contained 17 mutations that are expected to yield a translational change (14 nonsynonymous and 3 deletions), with several mutations located in the S gene. This specific lineage, which necessitates the use of sequencing-based analysis methods for its definitive ascertainment, emphasizes the need for SARS-CoV-2 sequencing programs to detect and monitor ongoing evolutionary patterns.

During the course of the SARS epidemic in 2003–2004, a number of mutations and deletions in *orf8* became prevalent in the population [40–42]. A 29-nucleotide deletion in SARS-CoV *orf8* was observed in nearly all cases diagnosed during the middle and end of the outbreak, and complete or almost complete deletions of *orf8* [40–42]. Evidence from our pangenome study suggests that SARS-CoV-2 *orf8* shows evidence of evolutionary divergence with less than 10% of all bases sharing an anchor 25-mer sequence (Fig. 2). Notably, we observed six total mutations between *orf7* and *orf8* in our clinical sample set. Three were nonsynonymous mutations (27874 C>T in samples P144 and P146, 27925 C>T in sample P138, 27970 C>T in P132) and are expected to result in a change from threonine to isoleucine (Additional file 1; Table S9). It will require additional studies to determine the consequences of these changes on viral fitness and disease, along with discovering evidence of positive selective pressure on this region of the viral genome.

Currently, there are a number of methods for detecting SARS-CoV-2 [43–45]. Quantitative reverse transcription polymerase chain reaction (RT-qPCR) is the most widely used SARS-CoV-2 diagnostic test and is considered the “gold standard” for detection due to its high specificity [44]. RT-qPCR diagnostic tests are based on the detection of viral nucleic acid from SARS-CoV-2 in respiratory specimens (such as nasopharyngeal and oropharyngeal swabs, sputum, and bronchoalveolar lavage fluid) collected from individuals suspected of COVID-19 infection. Serological antibody tests detect IgM and IgG generated in response to SARS-CoV-2 from blood samples. Antibody tests can provide information regarding current and previous SARS-CoV-2 infection, as well as potential immunity [46]. Neither serological tests nor RT-qPCR assays provide information about the viral sequence, the potential emergence of new strains or viral fingerprint patterns that are associated with transmission within a population. The role of viral sequencing is thus becoming increasingly important in diagnostic testing and disease control.

The increasing availability of SARS-CoV-2 genome sequences provides biomedical and clinical researchers with a rich resource for investigating pandemic spread. However, traditional alignment-based analysis techniques lead to challenges when applied on a scale of thousands of genome sequences. Our analysis is independent from coordinate systems of any given genome as a SARS-CoV-2 reference. Comparisons of viral genome assemblies scale exponentially as the number of samples increases. In contrast, our k-mer counting approach for identifying novel pangenome features scales linearly and therefore is computationally efficient. This approach enabled us to rapidly survey viral pangenome features such as genome conservation and mutation signatures from thousands of viral samples.

We developed a robust deep sequencing assay to ascertain quasispecies mutations and generate mutation signatures. We targeted the SARS-CoV-2 genome using primers from highly conserved regions based on our pangenome analysis. In the future, these same conserved primers can be used to detect novel mutations in additional viral regions. By virtue of designing primers in conserved regions, we expect that our assay will be able to amplify most strains of SARS-CoV-2, including regions of the viral genome that may be subject to increased selective pressure as vaccines become readily available to the global population. Notably, when performing the same type of k-mer analysis to primer sets used in the ARTIC network’s amplicon sequencing assay, we observed that only 57% of the primers appeared in at least 99% of the 75,681k GISAID dataset. This indicates that genomic variations observed in SARS-CoV-2 may both impact the performance and binding of primers as well as obscure mutations missed due to imperfect primer annealing and amplification during PCR.

This method also has the potential to be used for identifying recombination sites among viral genomes. By investigating unique k-mers in the SARS-CoV-2 genome, we may be able to identify recombination events that are representative of novel sequences. One limitation posed by short-read sequencing is that such structural changes in the viral genome may be less obvious given the length of the reads. The use of long read sequencing may overcome this issue. Another challenge is that a novel sequence breakpoint and new k-mers may be less readily identified when they occur at low frequency in a sample. These topics should be taken as considerations for the design and implementation of future studies.

We restricted the number of amplicons to improve the multiplexed amplification of viral genome sequences. However, one limitation of this deep sequencing approach is that coverage is restricted to approximately 40% of the viral genome. As a result, not all mutations present in a viral sample will be detected. When examining named SARS-CoV-2 strains, we observed three strains (20A, 20B, and 20C) out of twelve that could not be distinguished from each other using our designed assay. To overcome this issue, future iterations of assay design will expand the number of amplicons for broader coverage while maintaining primers in conserved sequences by leveraging the primer table that we have created. Thus, based on our pangenome analysis, there are opportunities to target additional mutation-prone sequences as they appear in the population. Previous studies have demonstrated the dynamic nature of mutation accumulation in the SARS-CoV-2 genome and their successive propagation throughout the human population [39]. When considering previously characterized SARS-CoV-2 lineages as well as future selective pressures due to both ongoing pandemic spread and vaccination, it will behoove future sequencing approaches to incorporate a flexible workflow to best adapt to novel changes in the viral genome. The primer design framework that we have developed for our sequencing approach both accomplishes this within our own sequencing workflow and facilitates adaptability for other researchers investigating the SARS-CoV-2 genome.

Our sequencing assay possesses important operational advantages compared to other molecular detection methods. Following sample processing, numerous individual specimens can be pooled during library preparation and maintain their unique identity. Identification of individual samples relies on assignments from DNA-based sample barcodes. Another advantage is the use of a library normalization procedure that eliminates the need for library balancing, greatly simplifying the sequencing library preparation workflow. For small genomes like SARS-CoV-2, one can sequence tens of thousands of samples in single sequencing run depending on the capacity of the sequencer. This scalability feature makes the analysis of large numbers of samples feasible compared to other assays that require samples to be maintained in individual wells throughout the entire sequencing workflow. The operational scalability of NGS also enables one to conduct large-scale population screening with the potential for significant cost reduction compared to other disease detection and diagnostic methods. In our study, we estimate that the total cost is approximately $20 per sample. As the number of samples increases, the use of higher capacity sequencing instruments will shift most of the cost burden to sample preparation; we estimate the total reagent cost could be lowered to $13 per sample. This is in the same range as the ARTIC sequencing workflow [47] and is substantially cheaper than studies utilizing shotgun approaches [15].

## Conclusions

Overall, the characterization of SARS-CoV-2 genetic variation provides insight into the paths of transmission and selection processes that may influence infection rates. Thus, viral mutations provide genetic fingerprints that cover a range of frequencies, such as the dominant species commonly observed among infected populations to less prevalent quasispecies that are limited to an individual or small number of patients. Analysis of lower frequency viral fingerprints may provide a way of conducting molecular contact tracing.

## Supplementary Information

**Additional file 1:.** Figure S1. Metadata k-mer indexing for mutations. Table S1. Genome assemblies included pangenome k-mer study. Table S2. K-mer filter criteria for primer design and selection. Table S3. Accession numbers of genomes included in in silico cross-reactivity analyses. Table S4. Multiplexed PCR primers used for amplicon generation and subsequent Illumina sequencing. Table S5. Concentrations of serially diluted contrived SARS-CoV-2 samples used for analytical sensitivity and specificity testing. Table S6. Strain-specific relative fractions of admixed SARS-CoV-2 contrived samples used for validaton of k-mer based analysis. Table S7. Strain-specific mutations detected from admixed SARS-CoV-2 contrived samples. Table S8. Cycle threshold (C_T_) values of externally tested clinical SARS-CoV-2 samples used for sequencing. Table S9. Expected translational changes from nonsynonymous mutations in orf7a and orf8 from clinical samples.

**Additional file 2:.** Table S10. The GISAID identities of 3968 SARS-CoV-2 genome assemblies. Table S11. The GISAID identities of 75,681 SARS-CoV-2 genome assemblies.

**Additional file 3:.** Table S12. The mutation landscape of 3968 SARS-CoV-2 genome assemblies. Table S13. The mutation landscape of 75,681 SARS-CoV-2 genome assemblies.

**Additional file 4:.** Table S14. Sequencing coverage metrics.

**Additional file 5:.** Acknowledgements to originating laboratories and submitting laboratories to GISAID.

## Data Availability

Sequence data is available at the National Institutes of Health’s Sequence Read Archive (SRA) at the URL listed below [35]. https://www.ncbi.nlm.nih.gov/bioproject/PRJNA663917. Analysis scripts are available at our GitHub URL listed below [24]. https://github.com/compbio/sars-cov-2-mutation-fingerprints. K-mer counts, candidate primer sequences, and processing scripts are also available at the URL listed below [48]. https://dna-discovery.stanford.edu/research/web-resources/sars-cov-2-mutation-fingerprints.

## Abbreviations

CDC: Centers for Disease Control and Prevention
cDNA: Complementary DNA
EUA: Emergency Use Authorization
FDA: Food and Drug Administration
GISAID: Global Initiative on Sharing All Influenza Data
gnomAD: Genome Aggregation Database
kb: Kilobase
NCBI: National Center for Biotechnology Information
NGS: Next-generation sequencing
nsp: Non-structural protein
nt: Nucleotide
ORF: Open reading frame
RdRp: RNA-dependent RNA polymerase
RT: Reverse transcription
ViPR: Virus Pathogen Resource
