# Supplementary material for: Profiling SARS-CoV-2 mutation fingerprints that range from the viral pangenome to individual infection quasispecies
Source: Genome Med. 2021 Apr 19;13:62. doi: 10.1186/s13073-021-00882-2 (PMC8054698; doi:10.1186/s13073-021-00882-2)
Supplement: Supplementary file 1 — Additional file 1:. Figure S1. Metadata k-mer indexing for mutations. Table S1. Genome assemblies included pangenome k-mer study. Table S2. K-mer filter criteria for primer design and selection. Table S3. Accession numbers of genomes included in in silico cross-reactivity analyses. Table S4. Multiplexed PCR primers used for amplicon generation and subsequent Illumina sequencing. Table S5. Concentrations of serially diluted contrived SARS-CoV-2 samples used for analytical sensitivity and specificity testing. Table S6. Strain-specific relative fractions of admixed SARS-CoV-2 contrived samples used for validaton of k-mer based analysis. Table S7. Strain-specific mutations detected from admixed SARS-CoV-2 contrived samples. Table S8. Cycle threshold (CT) values of externally tested clinical SARS-CoV-2 samples used for sequencing. Table S9. Expected translational changes from nonsynonymous mutations in orf7a and orf8 from clinical samples. [file 13073_2021_882_MOESM1_ESM.docx]

# SUPPLEMENTARY MATERIALS

# TITLE

Profiling SARS-CoV-2 mutation fingerprints that range from the viral pangenome to individual infection quasispecies

**Authors**

Billy T. Lau^1,2, †^, Dmitri Pavlichin^1,†^, Anna C. Hooker^1,†^, Alison Almeda^1^, Giwon Shin^1^, Jiamin Chen^1^, Malaya K. Sahoo^3^, ChunHong Huang^3^, Benjamin A. Pinsky^3,4^, HoJoon Lee^1^, Hanlee P. Ji^1,2^

^†^These authors contributed equally to this work.

**Institutions**

^1^Division of Oncology, Department of Medicine, Stanford University School of Medicine, Stanford, CA, 94305, United States

^2^Stanford Genome Technology Center West, Stanford University, Palo Alto, CA, 94304, United States

^3^Department of Pathology, Stanford University School of Medicine, Stanford, CA, 94305, United States

^4^Department of Medicine, Division of Infectious Diseases and Geographic Medicine, Stanford University School of Medicine, Stanford, CA, 94305, United States

**Corresponding authors**

Hanlee P. Ji Email: [genomics_ji@stanford.edu](mailto:genomics_ji@stanford.edu)

HoJoon Lee Email: [hojoon@stanford.edu](mailto:hojoon@stanford.edu)

Division of Oncology, Department of Medicine – Stanford University School of Medicine

269 Campus Drive, CCSR 1120, Stanford, CA 94305-5151


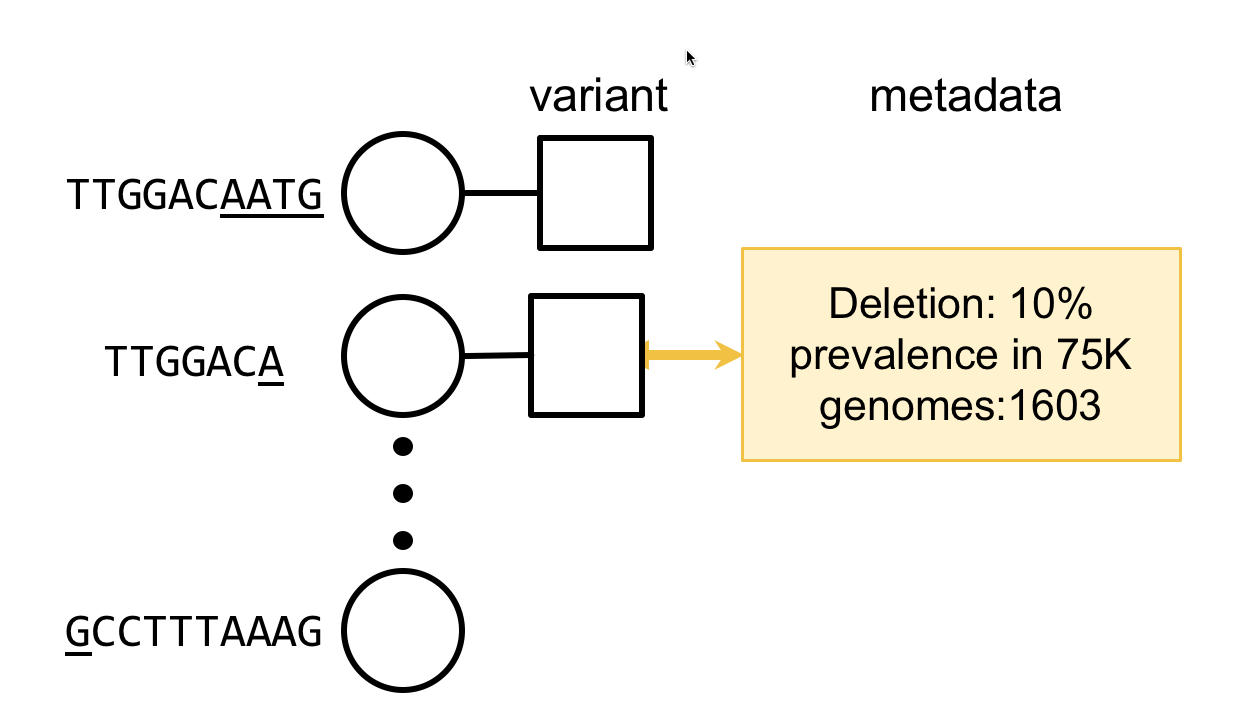


**Figure S1. Metadata kmer indexing for mutations.** We incorporated metadata annotating mutation features from the k-mer derived from individual SARS-CoV-2 genomes present in GISAID. This metadata includes the nature of the mutation and its frequency among the sample set.

**SUPPLEMENTARY TABLES**

**Table S1.** Genome assemblies included pangenome k-mer study.

**Table S2.** K-mer filter criteria for primer design and selection.

**Table S3.** Accession numbers of genomes included in *in silico* cross-reactivity analyses.

**Table S4.** Multiplexed PCR primers used for amplicon generation and subsequent Illumina sequencing.

|  |  | **Forward primers** | | | **Reverse primers** | | |  |
| --- | --- | --- | --- | --- | --- | --- | --- | --- |
| **Primer pair #** | **Target** | **Genomic coordinates** | | **Sequence** | **Genomic coordinates** | | **Sequence** | **Amplicon length (kb)** |
|  |  | **Start** | **End** |  | **Start** | **End** |  |  |
| 1 | SARS-CoV-2 | 1821 | 1845 | GTGCCTGGAATATTGGTGAACAGAA | 3048 | 3072 | CAATCACCTTCTTCTTCATCCTCAT | 1.252 |
| 3 | SARS-CoV-2 | 7450 | 7474 | AAGTTATGTGCATGTTGTAGACGGT | 8495 | 8519 | TAACAACTTGTCTAGTAGTTGCACA | 1.070 |
| 4 | SARS-CoV-2 | 9971 | 9995 | AAGGCTCTCAATGACTTCAGTAACT | 12446 | 12470 | TGGCTGCTGTTGTAAGAGGTATTAT | 2.500 |
| 6 | SARS-CoV-2 | 16264 | 16288 | TCACAGACTTCATTAAGATGTGGTG | 18267 | 18291 | ACGTACATGTCTTATAGCTTCTTCG | 2.028 |
| 7 | SARS-CoV-2 | 20996 | 21020 | GTGATTGTGCAACTGTACATACAGC | 23638 | 23662 | ACCAAGTGACATAGTGTAGGCAATG | 2.667 |
| 8 | SARS-CoV-2 | 26098 | 26122 | ATTGTTGATGAGCCTGAAGAACATG | 28480 | 28504 | ATTGGTGTTAATTGGAACGCCTTGT | 2.407 |
| 2 | human *RPP30* | 90889888 | 90889912 | CTTGTCATCGCATTTCTGTCATGTG | 90891190 | 90891214 | AGGTGGTCCTATAGATTTCAGAGGG | 1.327 |

**Table S5.** Concentrations of serially diluted contrived SARS-CoV-2 samples used for analytical sensitivity and specificity testing.

**Table S6.** Strain-specific relative fractions of admixed SARS-CoV-2 contrived samples used for validation of k-mer based analysis.

**Table S7.** Strain-specific mutations detected from admixed SARS-CoV-2 contrived samples.

**Table S8**. Cycle threshold (C_T_) values of externally tested clinical SARS-CoV-2 samples used for sequencing.

**Table S9.** Expected translational changes from nonsynonymous mutations in orf7a and orf8 from clinical samples.

| **Mutation** | 27641 C > T | 27670 G > T | 27874 C > T | 27925 C > T | 27970 C > T | 28144 T > C |
| --- | --- | --- | --- | --- | --- | --- |
| **Translational change** | S83L | V93F | T40I | T11I | T26I | L84S |
| **Protein** | ORF7a | ORF7a | ORF7b | ORF8 | ORF8 | ORF8 |
| **Wild type AA** | serine | valine | threonine | threonine | threonine | leucine |
| **Wild type AA polarity** | polar | nonpolar hydrophobic | polar | polar | polar | nonpolar hydrophobic |
| **Wild type AA MW (Da)** | 105.09 | 117.15 | 119.12 | 119.12 | 119.12 | 131.18 |
| **Mutant AA** | leucine | phenylalanine | isoleucine | isoleucine | isoleucine | serine |
| **Mutant polarity** | nonpolar hydrophobic | nonpolar aromatic | nonpolar hydrophobic | nonpolar hydrophobic | nonpolar hydrophobic | polar |
| **Mutant MW (Da)** | 131.18 | 204.23 | 131.18 | 131.18 | 131.18 | 105.09 |
| **Change in polarity** | from polar to nonpolar hydrophobic | from nonpolar hydrophobic to nonpolar aromatic | from polar to nonpolar hydrophobic | from polar to nonpolar hydrophobic | from polar to nonpolar hydrophobic | from nonpolar hydrophobic to polar |
| **Change in MW (Da)** | 26.09 | 87.08 | 12.06 | 12.06 | 12.06 | -26.09 |
| **Samples** | Pos_126 | Pos_139 | Pos_144, Pos_146 | Pos_138 | Pos_132 | Pos_145, Ctrl_001 |
| **GISAID frequency** *(n=75,681)* | 31 | 35 | 40 | 22 | 66 | 4655 |
| **GISAID %** | 0.040961404 | 0.046246746 | 0.052853424 | 0.029069383 | 0.08720815 | 6.150817246 |
